# Entropy optimization and heat flux analysis of Maxwell nanofluid configurated by an exponentially stretching surface with velocity slip

**DOI:** 10.1038/s41598-023-29137-3

**Published:** 2023-02-03

**Authors:** Saleem Nasir, Abdallah S. Berrouk, Asim Aamir, Zahir Shah

**Affiliations:** 1 Present Address: Mechanical Engineering Department, Khalifa University of Science and Technology, P.O. Box 127788, Abu Dhabi, United Arab Emirates; 2 Center for Catalysis and Separation (CeCas), Khalifa University of Science and Technology, P.O. Box 127788, Abu Dhabi, United Arab Emirates; 3 Department of Mechanical Engineering, Diponegoro University, Semarang, Indonesia; 4 Department of Mathematics, University of Lakki Marwat, Lakki Marwat, 28420 Khyber Pakhtunkhwa Pakistan

**Keywords:** Mathematics and computing, Nanoscience and technology

## Abstract

Hybrid nanofluids are extremely important in field of engineering and technology due to their higher heat transportation performance resulting in increased heat transfer rates. In the presence of thermal heat flux, the effect of a slanted MHD with velocity slip condition on a CNTs hybrid nanocomposite across a gradually extending surface is investigated. In present analysis, Maxwell nanofluid is embedded with SWCNT and MWCNT (single and multiple wall carbon nanotubes) nanoparticles. The nanomaterials transformation framework is obtained by employing Xue modified theoretical model. Various factors like dissipation, thermal radiations and Ohmic heat influences are adequately implemented in heat formulation. The physical features of thermodynamical mechanism of irreversibility are explored. The thermodynamics second law is used to produce the entropy optimization formulation. In addition, entropy is utilized to assess the energy aspects of a heat exchanger. Utilizing appropriate parameters, the model nonlinear PDEs are transformed to ODEs. The HAM technique is used to compute the solution of nonlinear ODEs. For both types of CNTs, the variations of entropy rate, Bejan number, velocity and temperature field versus key technical parameters is analyzed. The *Nu* and *C*_*f*_ computational result for both CNTs are examined in tabulated and chart form. Velocity is inversely proportional to magnetic and solid volume nanoparticle parameters. The *Br* and *Rd* accelerates *NG* and *Be* for both nanocomposites. Additionally, a comparison of the HAM result and the numerical result is validated.

## Introduction

The study of non-Newtonian fluids is essential in various manufactured fluids, including polymers, plastics, cellulose fibers, toothpaste, geological liquids, food products and many others. To determine the composition of these fluids, a variety of models have been developed. Generally, shear stress and rate are related in non-Newtonian fluids due to their nonlinearities. The equation of motion in such fluids has complicated nonlinear factors making it more difficult to evaluate. A variety of mathematical models reported in the literature to estimate the behavior of these fluids. The three major categories of these models are (i) Rate type models (ii) differential type models (iii) integral type models. Among the aforesaid categories of fluids, the rate and differential type fluids are more important. Fluids of the rate type are more important in investigations because they incorporate combined properties of storage and flexibility. As a result, the Maxwell fluid which is a subcategory of rate type fluids has been examined in present analysis. Maxwell^1^ initially introduced a simulation of these fluids to visualize the viscous behavior and flexibility of atmosphere. The temperature distribution utilizing Maxwell fluid with ramp velocities was investigated by Abdeljawad et al.^2^. Khan et al.^3^ examined a revolving disk with a Maxwell fluid polarized radiation flow. Megahed^4^ explored the process of energy transformation in combination with Maxwell fluid over a shrinking sheet. The impacts of convectional flow of Maxwell hybrid nanofluid across the channel were investigated by Hsiao^5^. Recently many scientists and scholars including Ahamd et al.^6^, Megahed^4^, Reddy et al.^7^ and Kumar et al.^8^ have successfully conducted comparable work with Maxwell hybrid nanofluids using various nanoparticles in different configurations.

The main focus has always been on achieving progress in establishing a fluid model that can help to improve the flow properties. The attempts do not end at introducing alternative model formulation, they also analyze current ideas and evaluate their applicability for the developed framework. Commonly, the MHD and viscous dissipation phenomena are two appropriate factors that should be considered among the available potential modifications to the fluid flow problem. It is universally accepted that a liquid along magnetohydrodynamic has the capacity to regulate flow regime and manipulate heat exchange in particular liquids. In the meantime, viscous dissipation occurs when fluid particles move and the viscosity of the liquid converts mechanical energy to heat energy. The given two actions are distinct from one another and can be analyzed separately. According to the literature the idea of MHD was identified in the equation of motion while viscous dissipation was established in the equation of heat. For a viscous motion of fluid Seth et al.^9^ numerically examined the combined impacts of MHD and heat generation. Mishra et al.^10^ found the occurrence of many computations when analyzing magnetohydrodynamic movement of flow of liquid including the impact of radiated heat and viscid dissipation across an expanding surface. According to Asimoni et al.^11^ the development of MHD in the direction of fluid motion was initially conducted in the context of 2D non-Newtonian nanofluid flow. Waini et al.^12^ published very new revelations on this topic in which the hybrid fluid is the primary motivation and the flow is considered to travel across a shrinking surface. A number of further materials on the MHD exploration are cited in the studies by Mabood et al.^13,14^.

Generally, one of the most interesting effects to be implemented is Joule heating because it empowers excellent control over the direction of hydromagnetic fluid movement which is important in many disciplines of science and technologies. Joule heating, often called as Ohmic heat is a method of converting electrical energy into heat energy which generates heat via resistant loss in the object. Furthermore, the idea of Joule heat is extensively and successfully used in the majority of electric, digital, and electronics equipment. Transferring an electrical potential to control losses in terms of current minimization is the most appropriate use of Joule heating. Many studies have examined at the movement of several MHD fluids with the Joule heating effect. Chakraborty et al.^15^ investigated the viscoelastic dissipation and Joule heating impacts on a flow of a EMHD fluid with a constant thermal gradient. Reddy et al.^16^ studied the movement of a Casson liquid and the cumulative effect of Joule heating and Hall in the inclined plane. Ramzan et al.^17^ evaluated the convection motion of a micropolar liquid due to the influence of slip effects and dissipating across an extending surface in the presence of linearly radiative heat. The works of Khan and Alzahrani^18^ and Shamshuddin et al.^19^ provide some more recent investigations on the Joule heating impact and MHD flow.

The advancement of nanotechnology has revolutionized our everyday lives. Several scientists and experts have spent considerable time to examining the flow behavior of various nanomaterials. It is due to its particular significance in a variety of technical, manufacturing and related disciplines. In fact, nanomaterials have a superior efficiency of thermal conductivity and play a key role in many heat transporting processes. Nanoliquid is a mixture of nano-sized from 1 nm to100 nm solid materials and conservatively functional substances that optimizes heat transport mechanisms. Nanoparticles have essential features that make them potentially important in a wide range of thermal management applications including cooling of engine, fuel cells, medicinal processes, residential freezers and so on. Generally, the volume fraction and thermophysical properties of nanomaterials affect the heat transport properties of nanomaterials. Initially Choi^20^ proposed using nanoparticles to progress the thermal conductivities of coolant (base fluid). Nanofluids have a wide range of uses, including centrifugal pumps, memory chips, air conditioning systems, tumor therapy, radiography, hybrid-powered vehicles, axially rotors compressors, treatment, battery storage, transport of nanodrug and a variety of other technologies. Using a computational method Ghosh and Swati^21^ established dual solutions of nanoliquid movement via an increasingly shrinking sheet involving thermal flux impacts. Lately some investigators such as Waqas et al.^22^ and Mabood et al.^23^ considered the study of nanofluid containing tiny nanoparticles in a variety of effects and geometries. By choosing to include superior and suitable nanoparticle ratio a contemporary class of nanoliquids called hybrid nanoliquids has recently been revealed. A hybrid nanofluid is comprised of two or more kinds of solid nanomaterials that are inserted into a single or composite conventional fluids. In almost all heat transmission disciplines such as microfluidic systems, transportation, production, microsystems and medicine, these brand-new hybrid nanocomposites have numerous potential applications. Devi and Devi^24^ computationally demonstrated 3D Al_2_O_3_ + Cu/H_2_O hybrid nanofluid movement along a stretchable sheet with various effects. Huang et al.^25^ designed a hybrid nanoliquid MWCNT*:*Al_2_O_3_ with a specific proportion of 1:2.5 by mixing Al_2_O_3_/H_2_O and MWCNT/H_2_O nano-fluids. In addition, several latest researches such as Nasir et al.^26,27^ and Khan et al.^28^ have concentrated on hybrid nano-liquids.

Entropy is a feature of any independent thermal system that specifies the degree of disturbance developed when thermal energy is converted into substantial work. From scientific point of view, it displays the microscopic properties of thermal systems and the assessment of unpredictability at atomic levels. The use of this principle is very significant in power station coolant, air conditioning units and steam turbines as well as heat pumps and energy stations. Bejan^29^ firstly investigated entropy minimization in convectional flow with heat flux. Jamshed^30^ examined the entropy effect in hybrid nanoparticles motion with thermal radiation. Hayat et al.^31^ discussed the role of molten heat transportation in a highly viscous MHD flow with entropy generation restricted by an extended surface. In literature various studies contain some significant analysis on the subject entropy generation such as Vatanparast et al.^32^, Mabood et al.^33,34^, Khan et al.^35^ and Berrouk et al.^36,37^.

### Significance analysis

The phenomena of entropy generation in a two-dimensional magnetohydrodynamic motion of hybrid nanoliquid subjected to an exponentially stretchable sheet is presented in this study. The following are the major motivations for conducting this research:

- The utilization of the entropy generation phenomena to hybrid nanoliquid movement subjected to an exponentially stretchable sheet is introduced.
- Thermal radiation, dissipation and Joule heating are all novel features of present study.
- The presence of novel features in velocity slip boundary conditions is also regarded unique.
- The investigation is conducted in the presence of a tilted magnetic field.
- Carbon nanotubes (SWCNTs and MWCNTs) as solid nanomaterials in a typical fluid (H_2_O) are used in this work while the behavior of liquid is considered Maxwell (non-Newtonian).
- The HAM approach is used to carry out the analytical computations and compared with numerical results.

On this point, it should be noted that certain studies have been published in literature on the flow of several nanofluid through an exponentially stretchable sheet. Moreover, the optimal flow of a hybrid nanoliquid employing viscous dissipation, Joule heating, and entropy has not yet to be explored. The findings of present analysis have significance in heat exchanger, chemical mechanisms, technological advancements, medical and engineering uses like electronics, heat transport, nuclear power stations and many other areas. The impact of several influential parameters on the physical explanation of hybrid nanofluid flow, thermal field and rate of entropy are examined. Computational outcomes of *C*_*f*_ and *Nu* against sundry different parameters are investigated.

## Problem formulation

**Figure 1:**
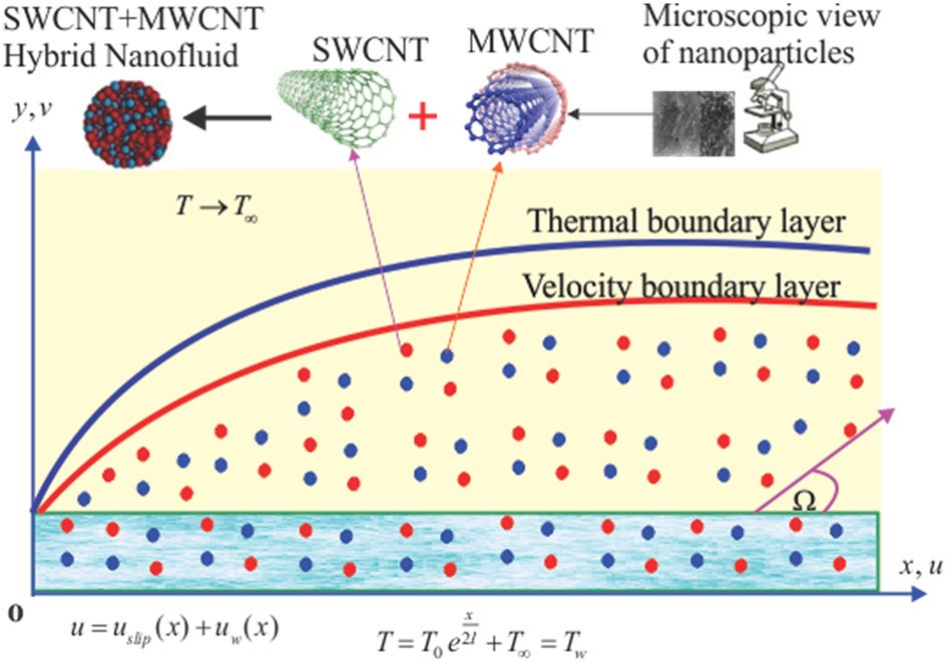
Flow configuration of model problem.

The two-dimensional time independent flow of water saturated by CNTs Maxwell hybrid nanofluid is taken through a continuously stretchy surface with velocity slip conditions. In current work the suspension is described by the Maxwell fluid model as a non-Newtonian liquid^7,8^. For nanomaterials two cases are considered, such as SWCNT/H_2_O nanofluid and SWCNT + MWCNT*/*H_2_O hybrid nanofluid. Furthermore, an inclined magnetic field is imposed in the direction of stretchable sheet with angle $\Omega$ and magnetic field strength $B(x) = B_{0} \,e^{\frac{x}{2l}}$, here $B_{0}$ uniform magnetic field strength^19^. While considering smaller values of magnetic Reynolds number the induced magnetic phenomena are neglected. The heat equation takes into account ohmic heating as well as viscous dissipation. Thermodynamic second law is explained in order to determine the rate of entropy generation. In the governing problem the fluid flow in *x*-axis as depicted in Fig. 1. Furthermore, $u_{w} (x) = u_{0} \,e^{\frac{x}{l}}$ is stretching velocity of sheet.

### Model equations

In light of the preceding limitations, the relevant boundary layer equations for 2D flow of the hybrid nanofluid in the presence of velocity slip boundary conditions are designed as^4,8,12,17^:

1 $$\frac{\partial u}{{\partial x}}\, + \,\frac{\partial v}{{\partial y}} = \,\,0,$$

2 $$u\frac{\partial u}{{\partial x}} + v\frac{\partial u}{{\partial y}} + \lambda_{1} \,\left( {u^{2} \frac{{\partial^{2} u}}{{\partial x^{2} }} + 2uv\frac{{\partial^{2} u}}{\partial x\partial y} + v^{2} \frac{{\partial^{2} u}}{{\partial y^{2} }}} \right) = \frac{{\mu_{Hbnf} }}{{\rho_{Hbnf} }}\frac{{\partial^{2} u}}{{\partial y^{2} }} - \frac{{\sigma_{Hbnf} }}{{\rho_{Hbnf} }}B^{2} \left( x \right)u\sin^{2} \Omega ,$$

3 $$\begin{gathered} \left( {\rho C_{p} } \right)_{Hbnf} \left[ {u\frac{\partial T}{{\partial x}} + v\frac{\partial T}{{\partial y}}} \right] = \left( {k_{Hbnf} + \frac{{16\sigma^{*} }}{{3k^{*} }}T_{\infty }^{3} } \right)\frac{{\partial^{2} T}}{{\partial y^{2} }} + \left\{ {\mu_{Hbnf} } \right.\left( {\frac{\partial u}{{\partial y}}} \right)^{2} - \lambda_{1} \left( {2u} \right.v\frac{\partial u}{{\partial x}}\frac{\partial u}{{\partial y}} \hfill \\ + v^{2} \left. {\left. {\left( {\frac{\partial u}{{\partial y}}} \right)^{2} } \right)} \right\} + \sigma_{Hbnf} B^{2} \,u^{2} \left( x \right), \hfill \\ \end{gathered}$$

Here Eq. (1) is the law of conservation of mass which indicates that mass is conserved when it leaves and enters a closed surface at the same rate. The nature of fluid flow is described by Eq. (2), which is a basic form of motion equations. Equation (3) is a formal variant of the energy equation that shows how temperature is dispersed near and across the surface. Also $u$ and $v$ are velocities components, $\sigma^{*}$ is symbolized “Stefan Boltzmann constant” and $k^{*}$ is denoted the Mean absorption coefficient. Equations (2) and (3) make it evident that the proposed non-Newtonian Maxwell model can be converted to a Newtonian model if $\lambda_{1} = 0$^4^. The $\sigma_{Hbnf}$—Electrical conductivity, $\mu_{Hbnf}$—Dynamic viscosity, $\rho_{Hbnf}$—Density, $\left( {\rho c_{p} } \right)_{Hbnf}$—Capacity of specific heat and $k_{Hbnf}$—Thermal conductivity are few characteristics of hybrid nanomaterials define as^7,8^.

4 $$\frac{{\sigma_{Hbnf} }}{{\sigma_{f} }} = 1 + \frac{{3\,\sigma_{SWCNT} \,\phi_{SWCNT} \, + 3\,\sigma_{MWCNT} \,\phi_{MWCNT} - 3\,\phi \,\sigma_{f} }}{{\sigma_{f} \,(2 + \phi ) + (1 - \phi_{SWCNT} )\,\sigma_{SWCNT} + (1 - \phi_{MWCNT} )\,\sigma_{MWCNT} }},$$

5 $$\frac{{\mu_{Hbnf} }}{{\mu_{f} }} = \frac{{\left( {1 - \phi_{MWCNT} } \right)^{ - 2.5} }}{{\left( {1 - \phi_{SWCNT} } \right)^{2.5} }},$$

6 $$\frac{{\rho_{Hbnf} }}{{\rho_{f} }} = \left( {1 - \phi_{MWCNT} } \right)\left( {1 - \phi_{SWCNT} } \right) + \frac{{\rho_{MWCNT} }}{{\rho_{f} }}\left( {1 - \phi_{MWCNT} } \right)\phi_{SWCNT} + \phi_{MWCNT} \frac{{\rho_{SWCNT} }}{{\rho_{f} }},$$

7 $$\frac{{\left( {\rho c_{p} } \right)_{Hbnf} }}{{\left( {\rho \,c_{p} } \right)_{f} }} = (1 - \phi_{SWCNT} )\,(1 - \phi_{MWCNT} ) + \phi_{SWCNT} (1 - \phi_{MWCNT} )\left( {\rho \,c_{p} } \right)_{MWCNT} + \phi_{MWCNT} \left( {\rho \,c_{p} } \right)_{SWCNT} ,$$

8 $$\frac{{k_{Hbnf} }}{{k_{nf} }} = (1 - \phi_{MWCNT} ) + 2\left( {\frac{{k_{SWCNT} }}{{k_{SWCNT} - k_{nf} }}} \right)k_{MWCNT} \log_{e} \left( {\frac{{k_{SWCNT} + k_{nf} }}{{k_{nf} }}} \right),$$

9 $$\frac{{k_{nf} }}{{k_{f} }} = (1 - \phi_{SWCNT} ) + 2\left( {\frac{{k_{MWCNT} }}{{k_{MWCNT} - k_{nf} }}} \right)k_{SWCNT} \log_{e} \left( {\frac{{k_{MWCNT} + k_{nf} }}{{k_{nf} }}} \right).$$

Also $\phi = \phi_{SWCNT} + \phi_{MWCNT}$. Here the subscribed *Hbnf, nf* and *f* are signifying hybrid, nano and base fluid respectively. SWCNT and MWCNT show single and multi-wall carbon nanotubes. For water (*Pr* = 6.2) at 20–25 °C, the thermos-physical features of SWCNT and MWCNT are displayed in Table 1^7,8^. Water is used as the primary fluid in this investigation. Aside from that, water has the highest specific heat making it an ideal cooling ingredient.

**Table 1:** Some thermo-physical features of CNTs and H_2_O^7,8^.

Physical properties	Nanoparticles		H_2_O
	SWCNT	MWCNT	
$k\;\left( {{\text{W}}/{\text{mK}}} \right)$	6600	3000	0.613
$c_{p} \;\left( {{\text{J/kg}}\,{\text{K}}} \right)$	425	796	4179
$\rho \;\left( {{\text{kg/m}}^{3} } \right)$	2600	1600	997.1
$\sigma \;\left( {\text{s/m}} \right)$	10^6^	10^7^	5.5 × 10^–6^

### Boundary conditions

The following is a description of the suitable flow conditions in the presence of velocity slip^4,19,21^:

10 $$\begin{aligned} & u = u_{slip} (x) + u_{w} (x),\,\,v = 0,\,\,T\, = T_{0} \,e^{\frac{x}{2l}} + T_{\infty } = T_{w} ,\,\,\,\,\,\,\,\,\,at\,\,\,\,\,y = 0, \hfill \\ & u \to 0,\,\,\,\,\,\,\,T \to T_{\infty } ,\,\,\,\,\,\,\,\,\,\,\,\,\,at\,\,\,\,\,y = \infty . \hfill \\ \end{aligned}$$

where $u_{slip}$ is the velocity slip component that varies with *x*. $T$ is temperature field, $T_{0}$, $T_{\infty }$ and $T_{w}$ are reference, ambient and stretching wall temperatures of sheet respectively^4^.

11 $$u_{slip} (x) = \Gamma \upsilon \left( {\mu \frac{\partial u}{{\partial y}} - \lambda_{1} \left( {2uv\frac{\partial u}{{\partial y}} + v^{2} \frac{\partial u}{{\partial y}}} \right)} \right),\,\,\,\,\,{\text{where}}\,\,\,\Gamma = \Gamma_{1} \,e^{\frac{x}{2l}} ,$$

Here $\Gamma_{1}$ is the starting value of velocity slip parameter.

### Transformation analysis

To transform the aforesaid PDEs in (1–4) in form of ODEs employing the variables as^21^.

12 $$\left. \begin{aligned} & u = u_{0} \,\,F^{\prime}\left( \xi \right)e^{\frac{x}{l}} ,\,\,v = - \left( {\frac{{\nu_{f} u_{0} }}{2l}} \right)^{\frac{1}{2}} e^{\frac{x}{2l}} \,\,\left\{ {F\left( \xi \right) - \xi \,\,F^{\prime}\left( \xi \right)} \right\},\, \hfill \\ & T = T_{\infty } + T_{0} \,e^{\frac{x}{2l}} \,\theta \left( \xi \right),\,\,\,\,\,\,\xi = y\left( {\frac{{u_{0} }}{{2\nu_{f} \,l}}} \right)^{\frac{1}{2}} e^{\frac{x}{2l}} \hfill \\ \end{aligned} \right\}$$

Equation (1) is simultaneously resolved after performing the preceding changes and other model equations take the form

13 $$\frac{{\varepsilon_{2} }}{{\varepsilon_{1} }}F^{\prime\prime\prime} - \delta_{m} \left( {\,F^{2} F^{\prime\prime\prime} + 4F^{{\prime}{^{3} }} - \xi \,F^{\prime\prime}F^{{\prime}{^{2} }} - 6F\,F^{\prime}\,F^{\prime\prime}} \right) - 2\,F^{{\prime}{^{2} }} + F\,F^{\prime\prime} - \frac{{\varepsilon_{3} }}{{\varepsilon_{1} }}M\,F^{\prime}\sin^{2} \Omega = 0,$$

14 $$\left( {\varepsilon_{4} + \frac{4}{3}Rd} \right)\theta^{\prime\prime} + Pr\varepsilon_{5} \left( {F\theta^{\prime} - F^{\prime}\,\theta } \right) + \varepsilon_{2} Br\,\left( {F^{{\prime\prime}{^{2} }} + \lambda_{1} \left( {2FF^{{\prime}{^{2} }} F^{\prime\prime} - F^{{^{2} }} F^{{\prime\prime}{^{2} }} } \right)} \right) + \varepsilon_{3} Br\,F^{{\prime}{^{2} }} = 0,$$

The boundary constraint looks like:

15 $$\left. \begin{aligned} & F^{\prime}\left( \xi \right) = \left( {\gamma F^{\prime\prime}\left( \xi \right) + \lambda_{1} \left( {2F\left( \xi \right)F^{{\prime}{2}} \left( \xi \right) - F^{2} \left( \xi \right)F^{\prime\prime}\left( \xi \right)} \right)} \right) + 1,F\left( \xi \right) = 0,\,\Theta \left( \xi \right) = 1\,{\text{at}}\,\xi = 0 \hfill \\ & F^{\prime}\left( \xi \right) \to 0,\,\,\,\,\,\Theta \left( \xi \right) \to 0\,,\Theta \to 0,\,\,\,\,\,\,\,\,\,\,\,\,\,\,\,\,\,\,\,\,\,\,\,{\text{at}}\,\,\,\,\xi \to \infty \hfill \\ \end{aligned} \right\}$$

The non-dimensional velocity and temperature components are $F\left( \xi \right)$ and $\Theta \left( \xi \right)$.

16 $$\varepsilon_{1} = \frac{{\rho_{hnf} }}{{\rho_{f} }},\,\,\,\,\,\varepsilon_{2} = \frac{{\mu_{hnf} }}{{\mu_{f} }},\,\,\,\,\,\varepsilon_{3} = \frac{{\sigma_{hnf} }}{{\sigma_{f} }},\,\,\,\,\,\varepsilon_{4} = \frac{{k_{hnf} }}{{k_{f} }},\,\,\,\,\,\varepsilon_{5} = \frac{{\left( {\rho C_{p} } \right)_{hnf} }}{{\left( {\rho C_{p} } \right)_{f} }},$$

Moreover, additional non-dimensional factors include

17 $$\left. \begin{aligned} & {\text{velocity}}\,\,{\text{slip}}\,\,{\text{parameter}}\, = \gamma = l\,\left( {\frac{2\Omega }{{\upsilon_{f} }}} \right)^{1/2} ,\,{\text{Maxwel}}\,{\text{parameter}} = \delta_{m} = \lambda_{1} \left( {\frac{{u_{0} }}{2\,l}} \right)e^{\frac{x}{l}} ,\, \hfill \\ & {\text{Prandtl}}\,\,{\text{number}} = \Pr = \frac{{\left( {\mu C_{p} } \right)_{f} }}{{k_{f} }},\,\,{\text{Magnetic}}\,\,{\text{parameter}} = M = \frac{{2\sigma_{f} B_{0}^{2} l}}{{\rho_{f} u_{0} }},\, \hfill \\ & {\text{Radiation}}\,\,{\text{parameter}} = R = \frac{{4\sigma^{*} T_{\infty }^{3} }}{{k^{*} \,k_{f} }},\,\,\,\,{\text{Eckert}}\,\,{\text{number}} = Ec = \left( {\frac{{u_{0}^{2} }}{{C_{p} \,T_{0} }}} \right)e^{{\frac{3x}{{2l}}}} ,\, \hfill \\ & {\text{Brinkman number}} = Br = PrEc. \hfill \\ \end{aligned} \right\}$$

## Modeling of entropy generation and Bejan number

The version of entropy generation for the model flow is^32,35^

18 $$\begin{aligned} S_{gen} & = \frac{1}{{T_{\infty }^{2} }}\left( {k_{Hbnf} + \frac{{16\sigma^{*} T_{\infty }^{3} }}{{3k^{*} }}} \right)\left( {\frac{\partial T}{{\partial y}}} \right)^{2} + \left\{ {\frac{{\mu_{Hbnf} }}{{T_{\infty } }}} \right.\left( {\frac{\partial u}{{\partial y}}} \right)^{2} - \lambda_{1} \left( {2u} \right.\frac{\partial u}{{\partial y}} + v^{2} \left. {\left. {\left( {\frac{\partial u}{{\partial y}}} \right)^{2} } \right)} \right\} \hfill \\ & \quad + \frac{{\sigma_{Hbnf} B^{2} \left( x \right)}}{{T_{\infty } }}u^{2} , \hfill \\ \end{aligned}$$

By employing the transformation Eq. (12) on Eq. (18) we get

19 $$N_{g} = \left( {\varepsilon_{4} + \frac{4}{3}Rd} \right)\alpha_{1} \theta^{{\prime}{^{2} }} + \varepsilon_{2} Br\,\left( {F^{{\prime\prime}{^{2} }} + \lambda_{1} \left( {2FF^{{\prime}{^{2} }} F^{\prime\prime} - F^{{^{2} }} F^{{\prime\prime}{^{2} }} } \right)} \right) + \varepsilon_{3} M\,Br\,F^{{\prime}{^{2} }} ,$$

20 $$\left. \begin{aligned} & {\text{The temperature ratio parameter}} = \alpha_{1} = \frac{{T_{0} }}{{T_{\infty } }}e^{\frac{x}{2L}} \hfill \\ & {\text{Entropy}}\,{\text{generation}}\,\,{\text{rate}} = N_{g} = \left( {\frac{{2l\,\,\nu_{f} S_{gen} \,\,T_{\infty } }}{{k_{f} \,\,T_{0} \,\,u_{0} }}} \right)\,\,\,e^{{\frac{3x}{{2l}}}} \hfill \\ \end{aligned} \right\}$$

We introduce additional significant exponent termed as $Be$(Bejan number)^30^, which is used to determine the relative importance of entropy production by heat transport.

21 $$Be = \frac{{N_{h} }}{{N_{h} + N_{f} + N_{m} }},$$

So, in dimensionless form

22 $$Be = \frac{{\alpha_{1} \,\left( {\varepsilon_{4} + \frac{4}{3}Rd} \right)\,\theta^{{\prime}{^{2} }} }}{{\left( {\varepsilon_{4} + \frac{4}{3}Rd} \right)\alpha_{1} \theta^{{^{{\prime}{2}} }} + \varepsilon_{2} Br\,\left( {F^{{\prime\prime}{^{2} }} + \lambda_{1} \left( {2FF^{{\prime}{^{2} }} F^{\prime\prime} - F^{{^{2} }} F^{{\prime\prime}{^{2} }} } \right)} \right) + \varepsilon_{3} M\,Br\,F^{{\prime}{^{2} }} }}.$$

Also, remember that when $Be = 1$, heat transport irreversibility phenomena take precedence and when $Be = 0$, entropy caused by fluid friction supersedes. Moreover, $Be = 0.5$, signifies the production of entropy since the frictional resistance of liquids and the heat transport are identical.

## Engineering quantities

The mathematical values of these quantities “$C_{f}$ and $Nu$” are described as

23 $$\left. {C_{f} = \frac{{2\mu_{Hbnf} }}{{u_{w}^{2} \,\rho_{Hbnf} }}\left( {\frac{\partial u}{{\partial y}}} \right)_{y = 0} ,\,\,\,\,\,\,Nu = \frac{x}{{k_{f} (T_{w} - T_{\infty } )}}\left( {k_{Hbnf} \frac{\partial T}{{\partial y}}} \right)_{y = 0} + \left( {q_{r} } \right)_{y = 0} } \right\},$$

Using (12) in (23), we get

24 $$\left. {\left[ {Re_{x} } \right]^{\frac{1}{2}} C_{f} = \frac{{\mu_{Hbnf} }}{{\mu_{f} }}F^{\prime\prime}\left( 0 \right),\,\,\,\,\left[ {Re_{x} } \right]^{{ - \frac{1}{2}}} Nu = - \left( {\frac{{k_{Hbnf} }}{{k_{f} }} + \frac{4}{3}Rd} \right)\theta^{\prime}\left( 0 \right)} \right\},$$

$Re_{x}$ is the local Reynolds number and stated as $Re_{x} = \frac{{x.\,u_{w} \left( x \right)}}{{\upsilon_{f} }}$.

## Research methodology

The purpose of this section is to use the HAM approach to generate a sequential solution of the flow problem. The proposed linear operators and their accompanying starting approximations $F_{0\,}$ and $\Theta_{0\,}$ for the hybrid nanofluid flow model boundary value problem is:

25 $$\left. {F_{0} (\xi ) = 1 - \exp \left( { - \xi } \right),\,\,\,\,\,\,\Theta_{0} (\xi ){\text{ = exp}}\left( { - \xi } \right)} \right\}$$

The following are the linear operators for velocity and temperature profiles, respectively:

26 $$\Im_{F} (F) = F^{{\prime} {\prime} {\prime}}\,\,\,\,\,\,\Im_{\theta } {(}\theta {) = }\theta ^{{\prime}{\prime}},$$

So, the expand form of $\aleph_{f}$ and $\aleph_{\Theta }$ are indicated as

27 $$\Im_{F} (\mathcal{R}_{1}^{*} + \mathcal{R}_{2}^{*} \xi + \mathcal{R}_{3}^{*} \xi^{2} ) = 0,\,\,\,\,\,\Im_{\Theta } (\mathcal{R}_{4}^{*} + \mathcal{R}_{5}^{*} \xi ) = 0\,,$$

where the arbitrary constant $\mathcal{R}_{k}^{*} (k = 1,2....,5)$.

## Results and discussion

**Figure 2:**
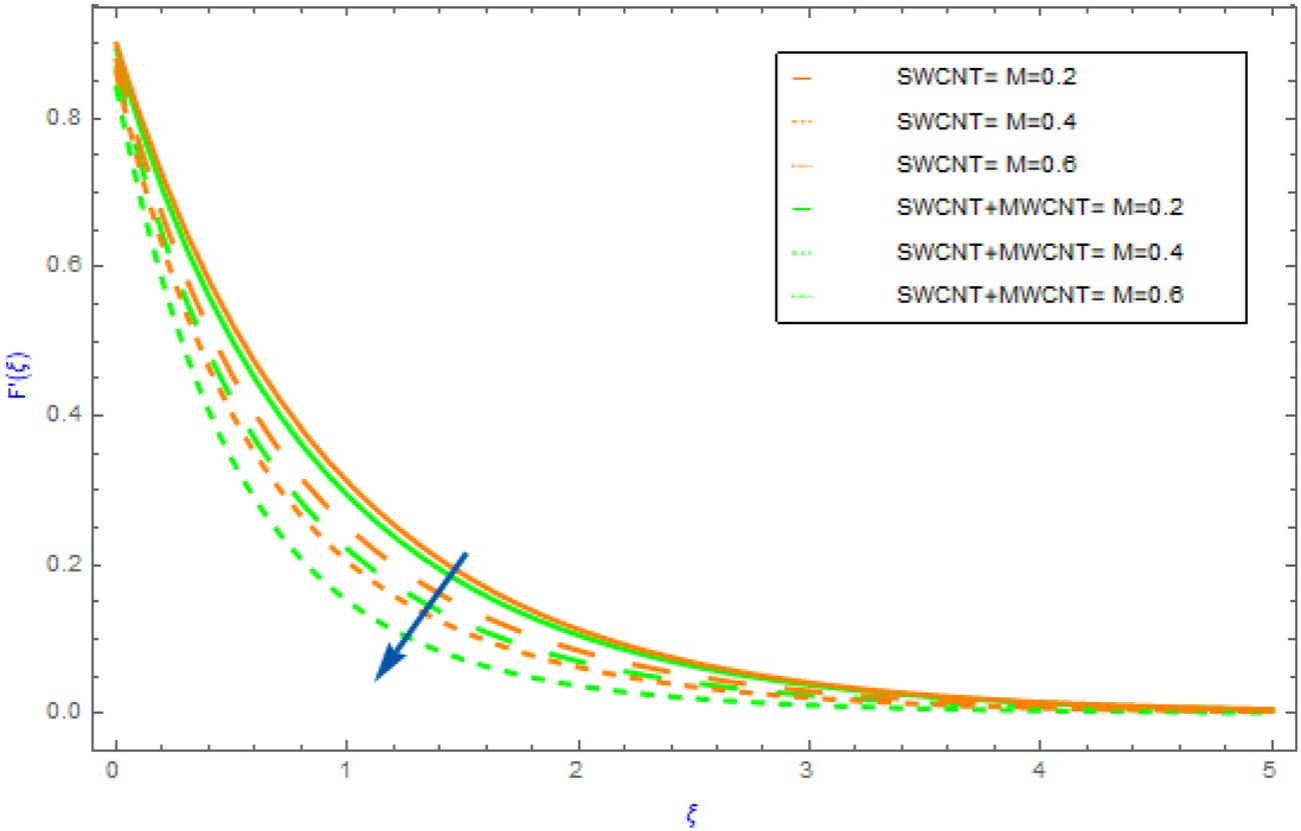
Impact of $M$ parameter on $F^{\prime}\left( \xi \right)$.

**Figure 3:**
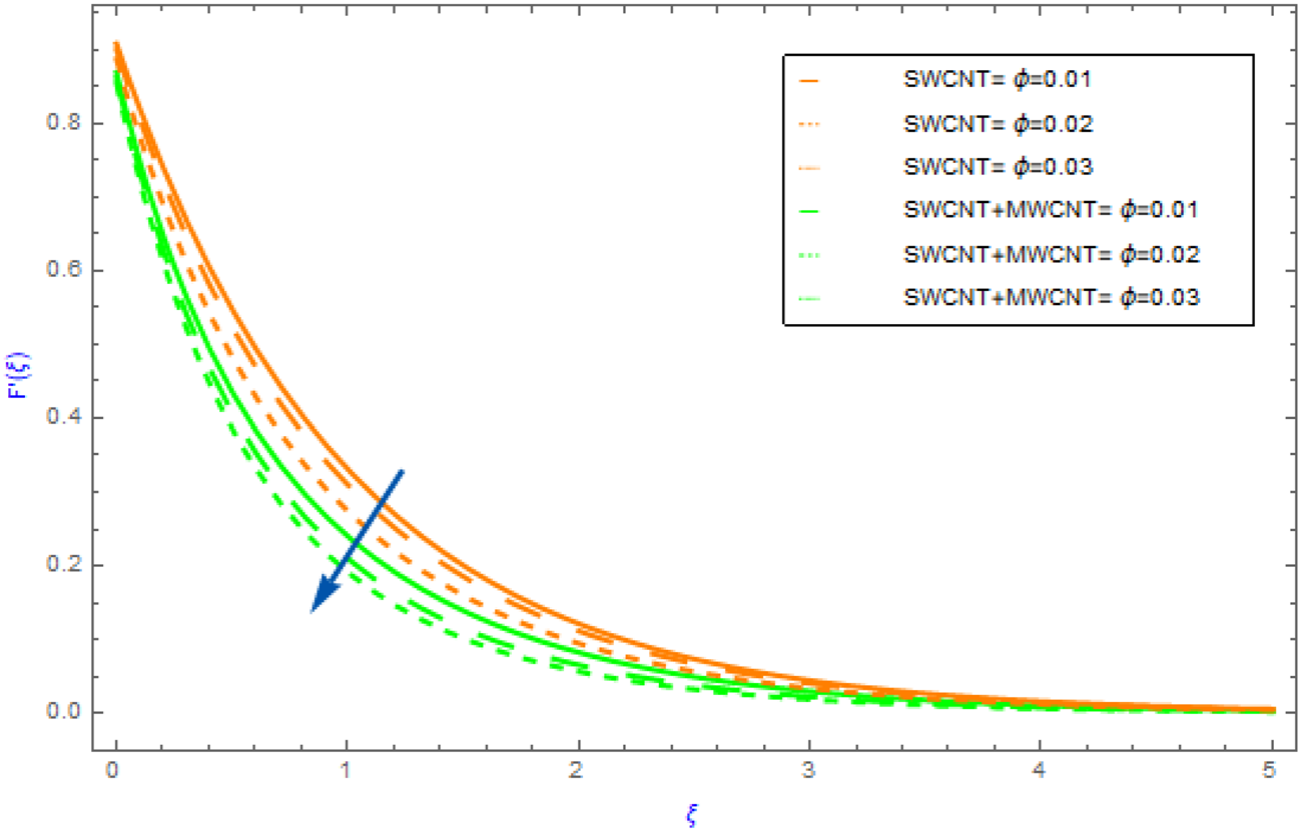
Impact of $\phi$ parameter on $F^{\prime}\left( \xi \right)$.

**Figure 4:**
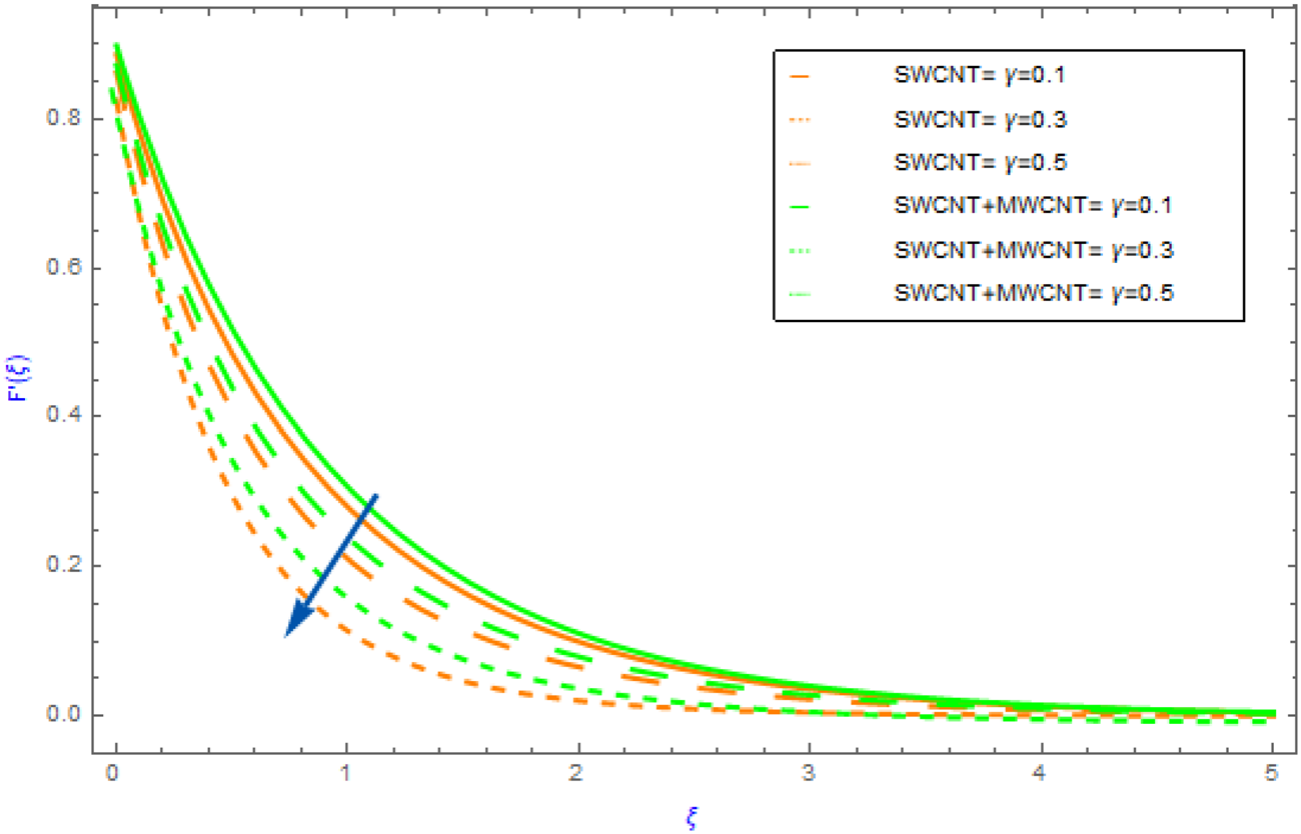
Impact of $\gamma$ parameter on $F^{\prime}\left( \xi \right)$.

**Figure 5:**
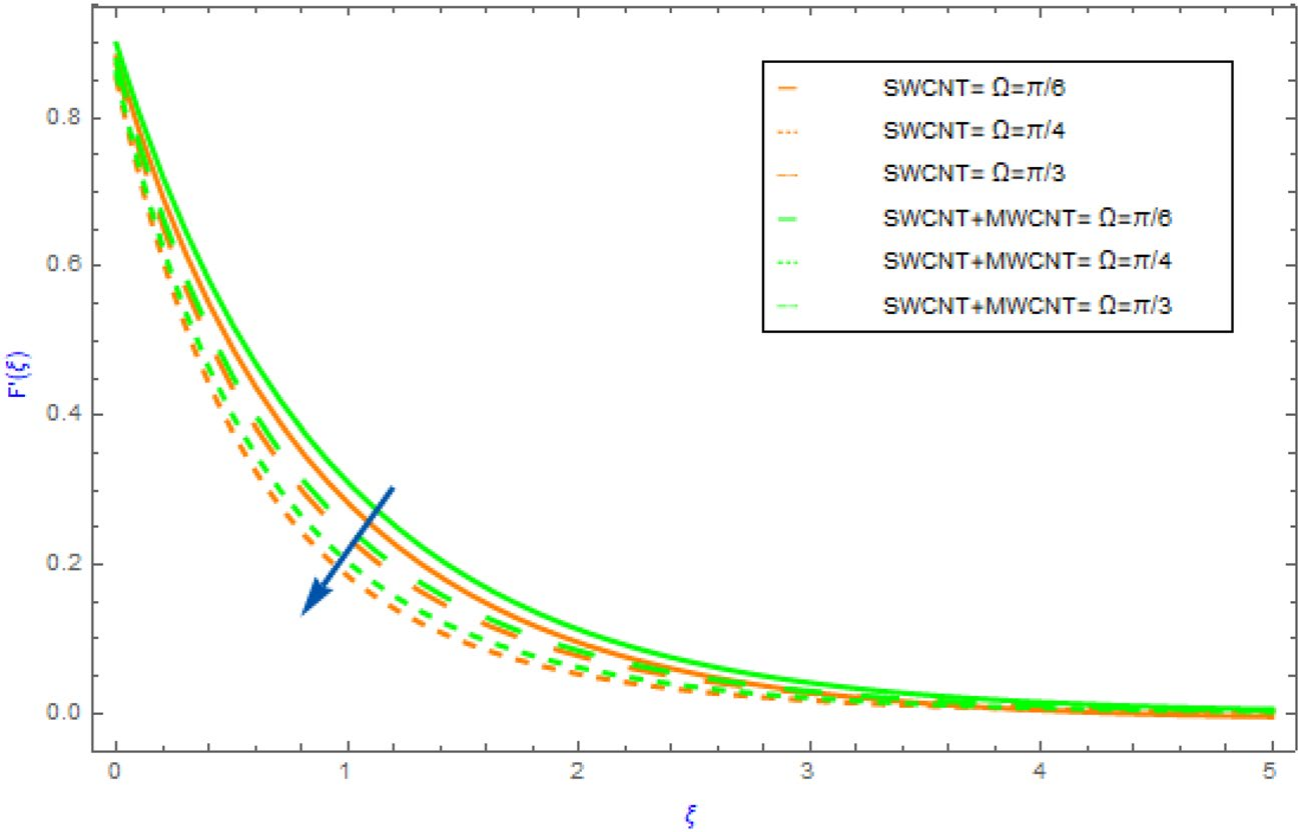
Impact of $\Omega$ parameter on $F^{\prime}\left( \xi \right)$.

**Figure 6:**
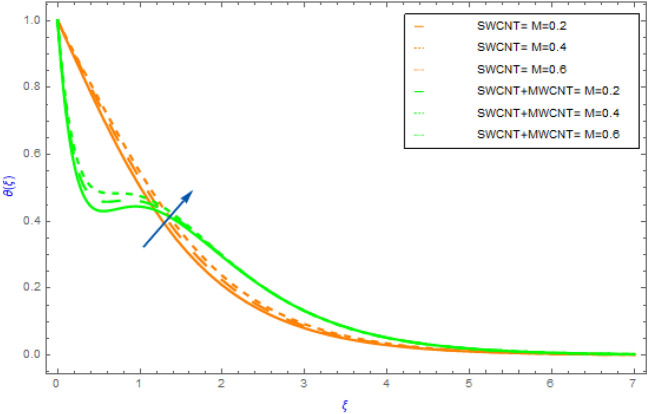
Impact of $M$ parameter on $\theta \left( \xi \right)$.

**Figure 7:**
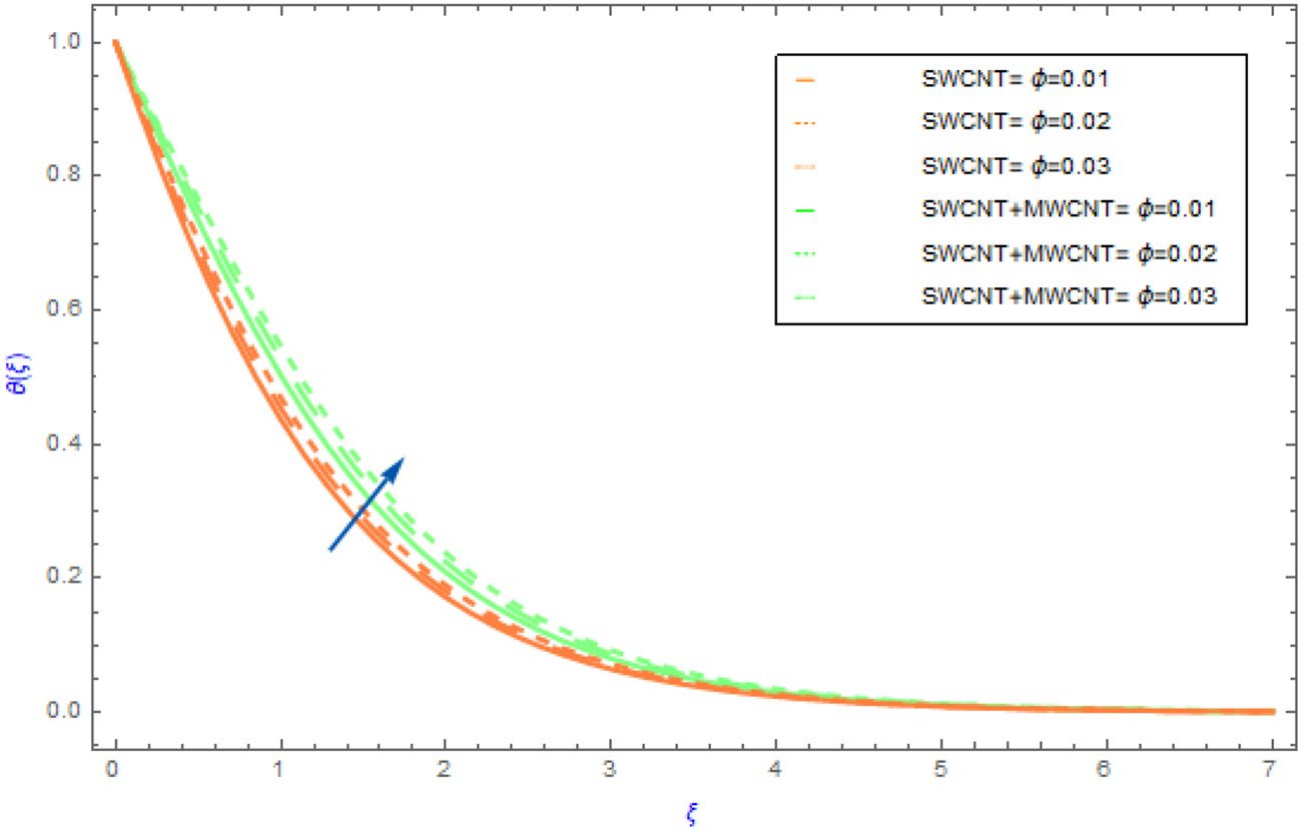
Impact of $\phi$ parameter on $\theta \left( \xi \right)$.

**Figure 8:**
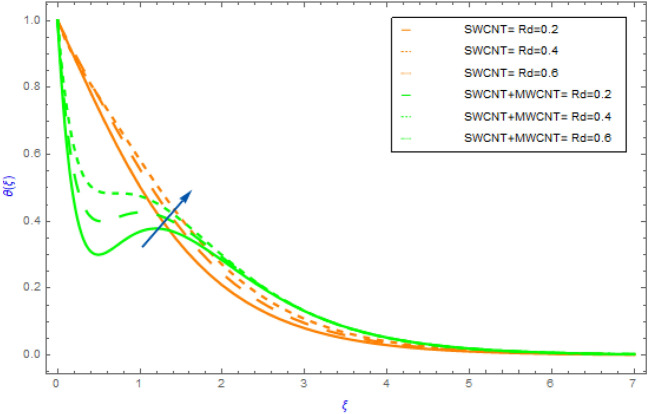
Impact of $Rd$ parameter on $\theta \left( \xi \right)$.

**Figure 9:**
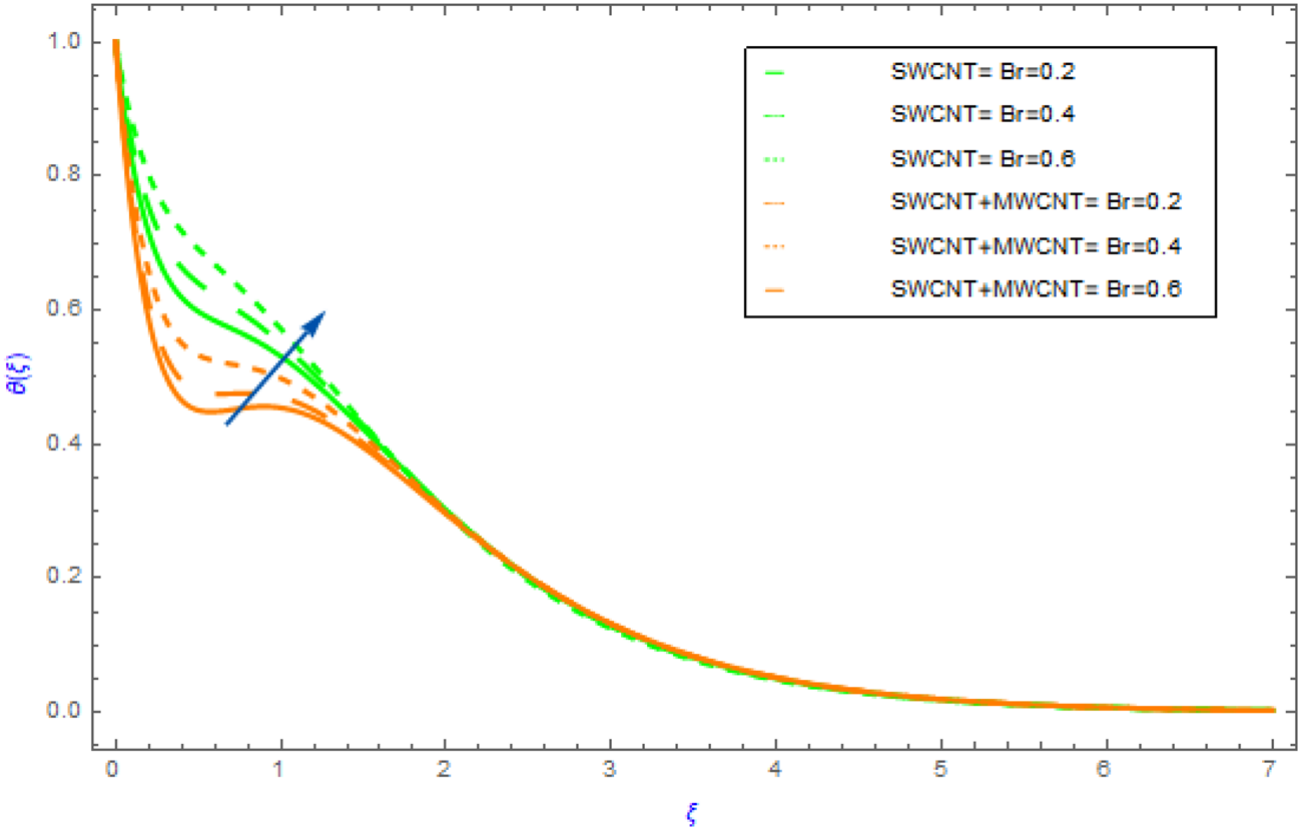
Impact of $Br$ parameter on $\theta \left( \xi \right)$.

**Figure 10:**
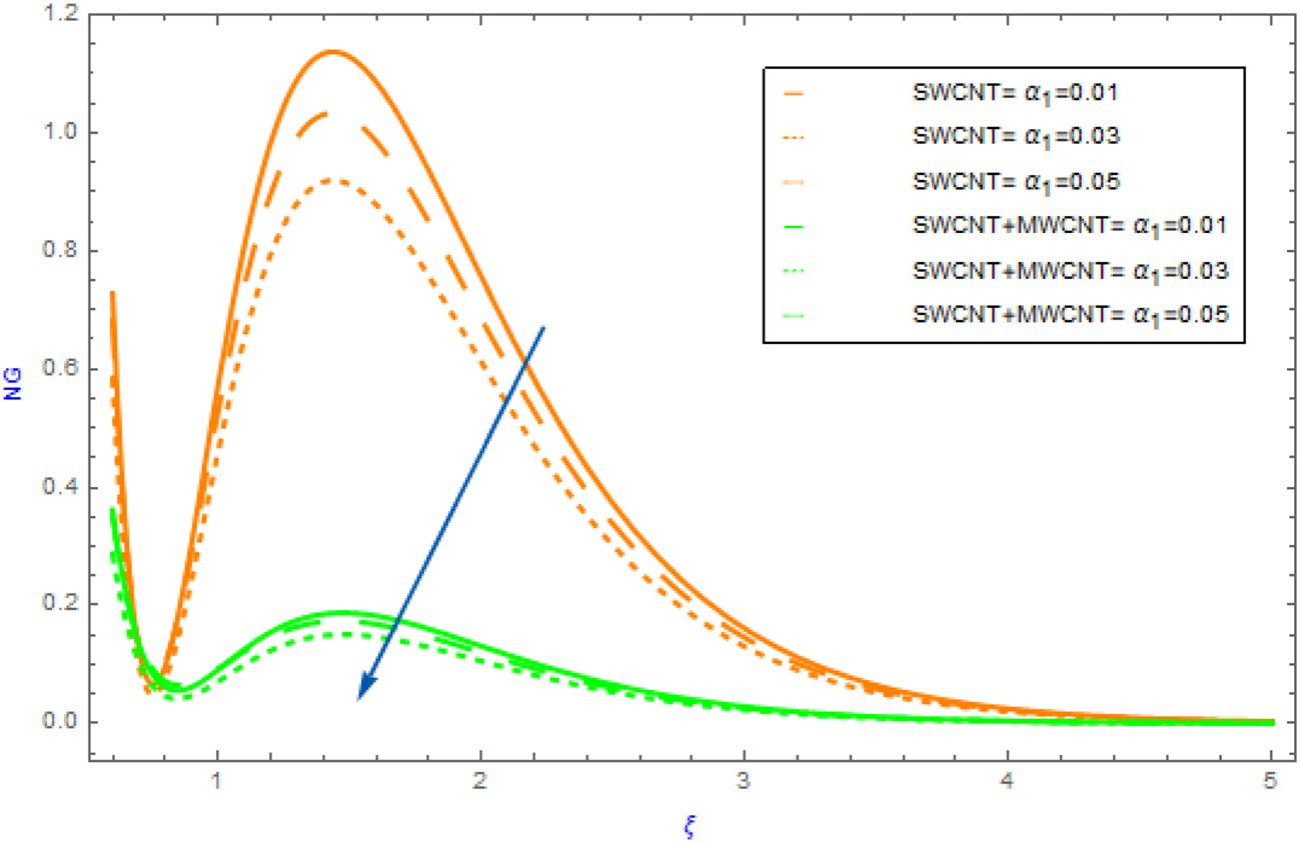
Impact of $\alpha_{1}$ parameter on $NG$.

**Figure 11:**
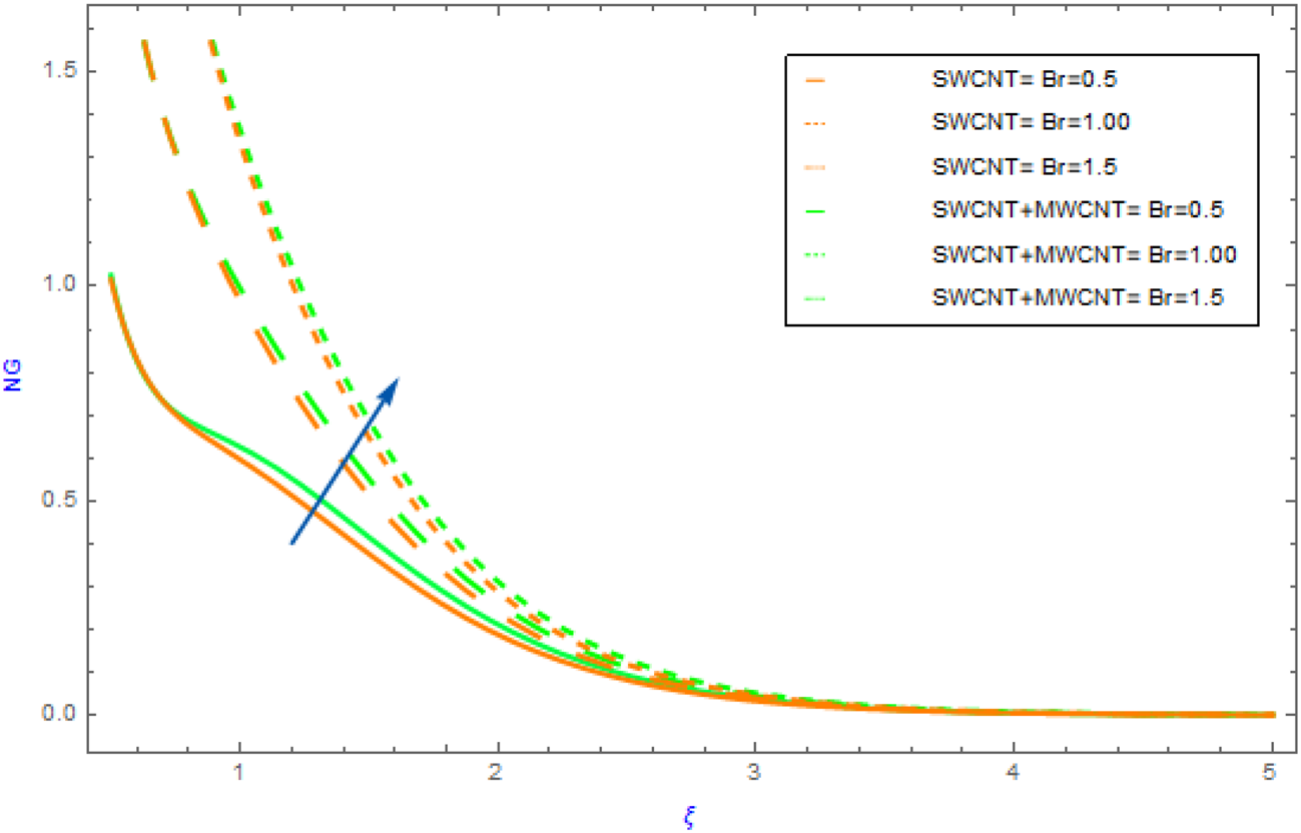
Impact of $Br$ parameter on $NG$.

**Figure 12:**
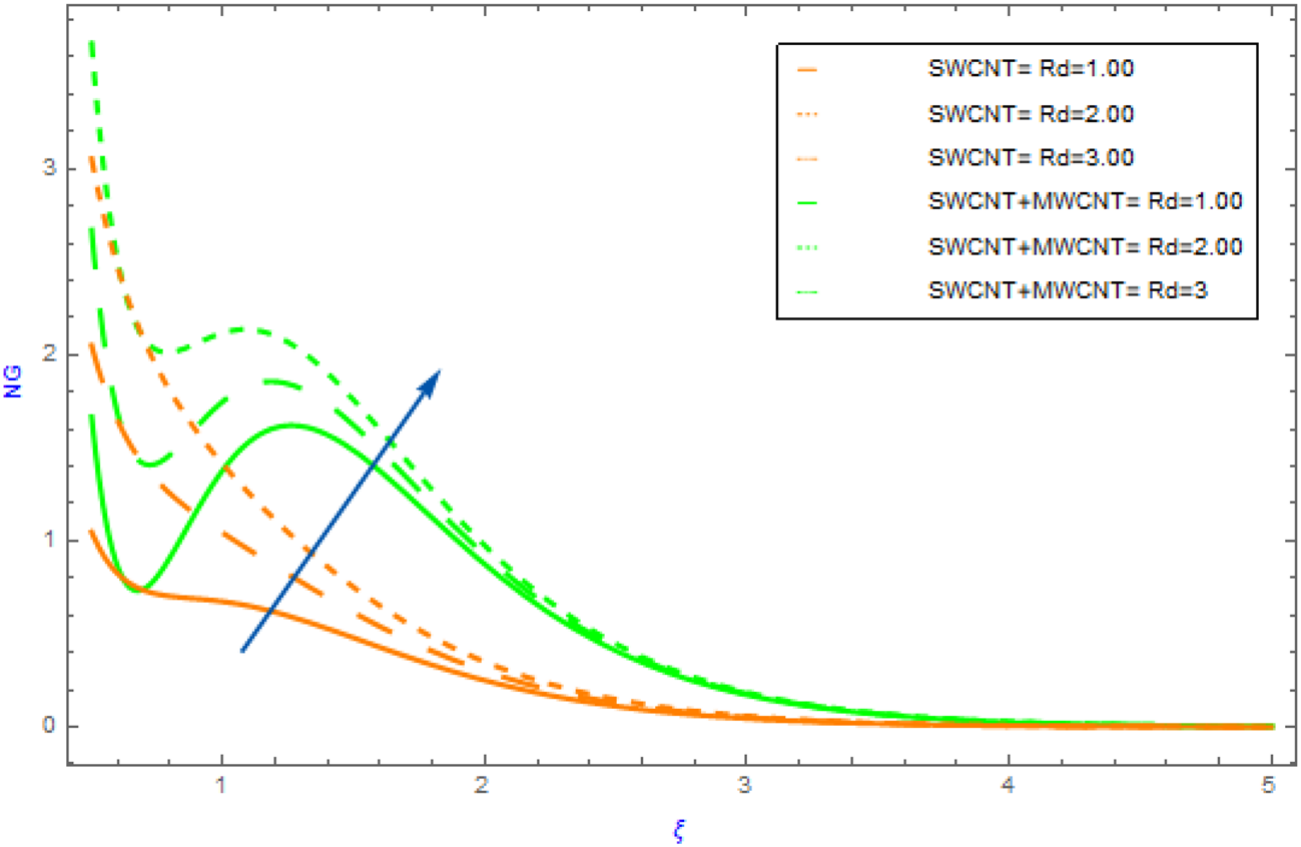
Impact of $Rd$ parameter on $NG$.

**Figure 13:**
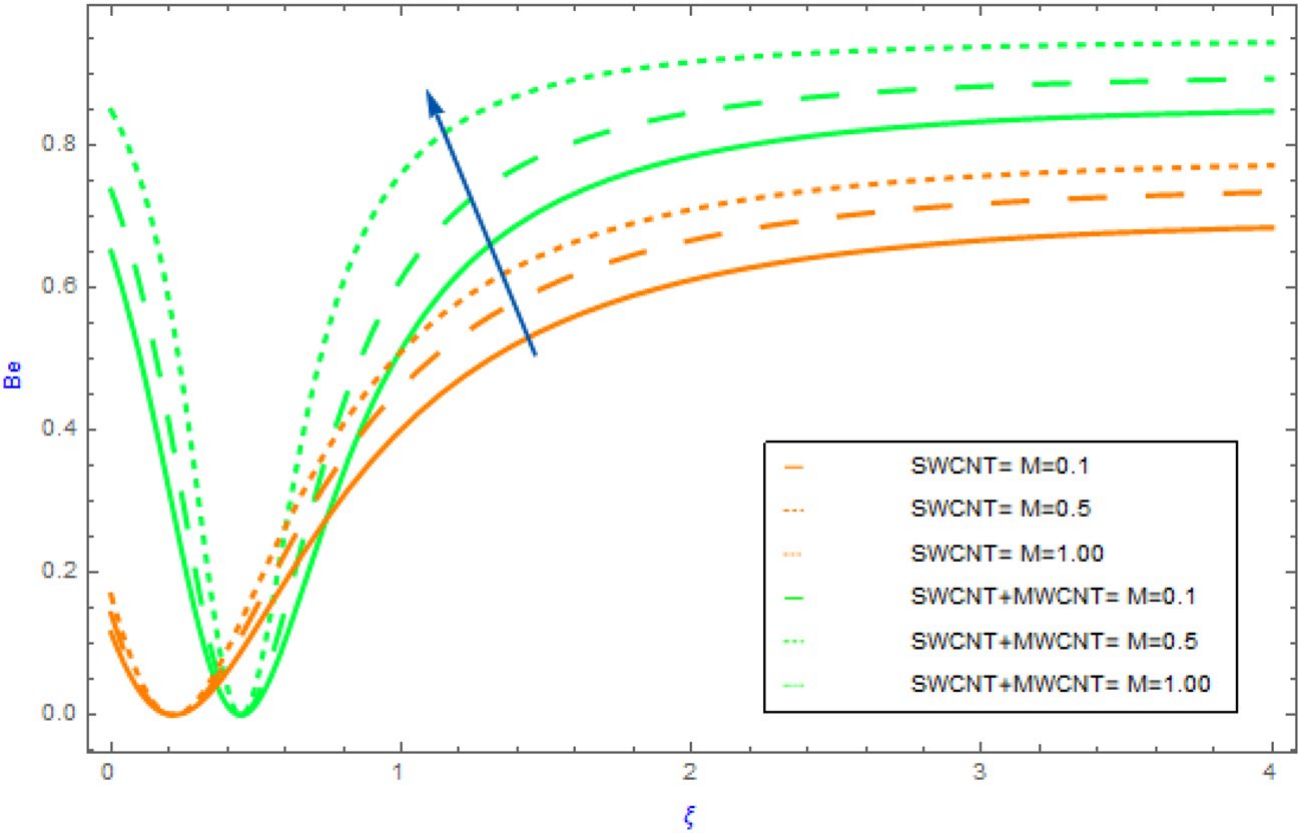
Impact of $M$ parameter on $Be$.

**Figure 14:**
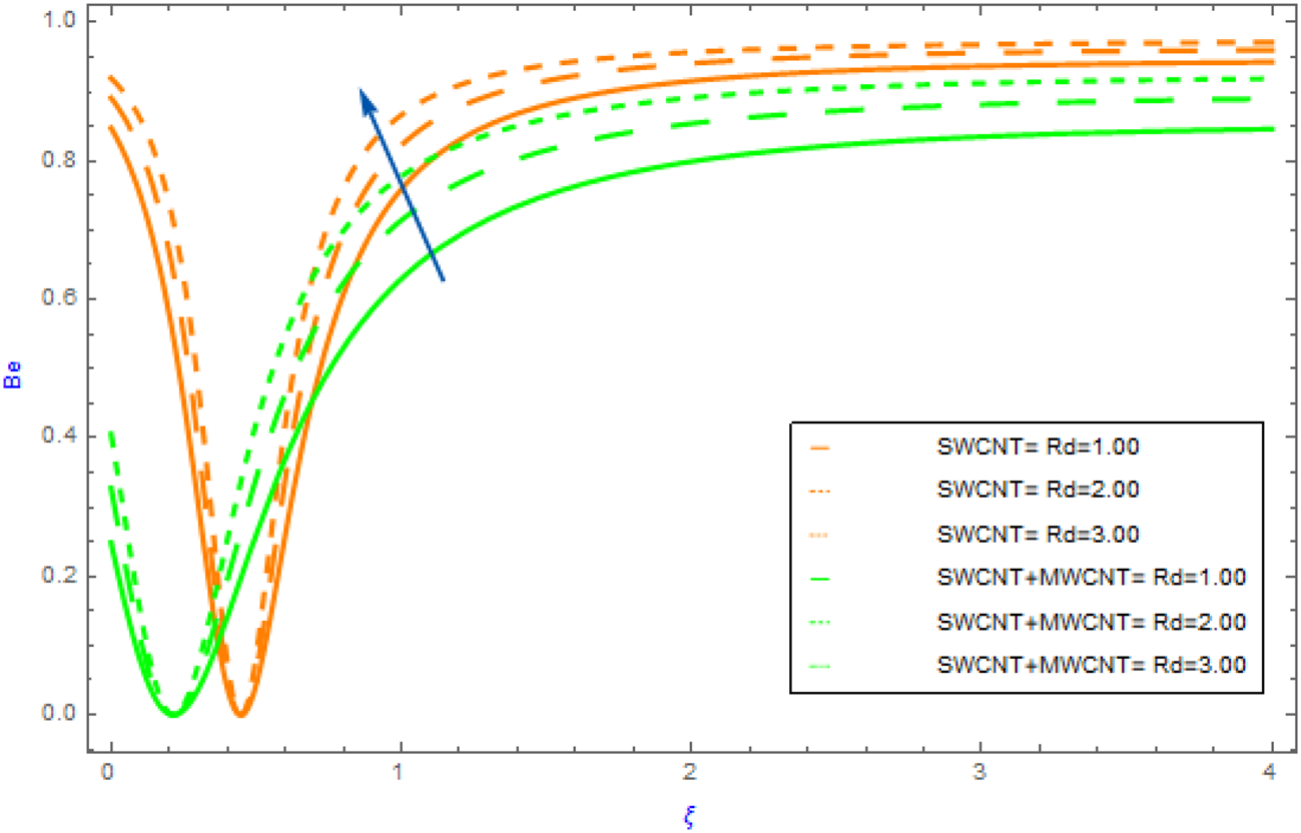
Impact of $Rd$ parameter on $Be$.

This portion is dedicated to the assessment of the material characteristics of immersion factors on quantities, such as $F^{\prime}\left( \xi \right)$(flow profile), $\theta \left( \xi \right)$(thermal profile), $NG$(entropy generation) and the $Be$(Bejan number) in regards to SWCNT/H_2_O unitary nanofluid and SWCNT + MWCNT/H_2_O hybrid nanofluid. All such findings are well explained and graphically presented (depicting in Figs. 2, 3, 4, 5, 6, 7, 8, 9, 10, 11, 12, 13, 14). The orange lines in these graphs depict the characteristics of SWCNT/H_2_O nanofluid, while the blue lines depict SWCNT + MWCNT/H_2_O behavior. Nusselt numbers and skin frictions are also estimated and examined see Tables 2 and 3. Table 1 also shows the physical and thermal properties of carbon nanotubes (SWCNTs and MWCNTs) and water (H_2_O). In our entire analysis, the values allocated to the physical variables are $M = 0.5,\,\,\gamma = 0.3,$ $\,\,\phi = 0.02,\,\,Pr = 6.2,\,\,Rd = 1.00,\,Br = 0.5$ and $\alpha_{1} = 0.03$.

**Table 2:** Different values of $\left[ {Re_{x} } \right]^{0.5} C_{f}$ for various parameters.

Fixed parameter	Parameter	Values	$\frac{{{\text{SWCNT}}/\text{H}_{2} \text{O}}}{{\left[ {Re_{x} } \right]^{0.5} C_{f} }}$	$\frac{{{\text{S + MWCNT}}/\text{H}_{2} \text{O}}}{{\left[ {Re_{x} } \right]^{0.5} C_{f} }}$
$$\begin{gathered} Br = 0.5,\gamma = 0.3,\delta_{m} = 0.2 \hfill \\ Pr = 6.2,Rd = 1,\Omega = \frac{\pi }{3},\alpha_{1} = 0.1. \hfill \\ \end{gathered}$$	$M$	0.5	1.6000613	1.6000793
		1.00	1.6604526	1.6605553
$$\begin{gathered} M = 0.5 = Br,\delta_{m} = 0.2,\,\phi = 0.02 \hfill \\ Pr = 6.2,Rd = 1,\Omega = \frac{\pi }{3},\alpha_{1} = 0.03. \hfill \\ \end{gathered}$$	$\gamma$	0.1	1.5991672	1.5900264
		0.2	1.6586403	1.6404022
$$\begin{gathered} M = 0.5 = Br,\gamma = 0.3,\delta_{m} = 0.2 \hfill \\ Pr = 6.2,Rd = 1,\alpha_{1} = 0.1. \hfill \\ \end{gathered}$$	$$\Omega$$	$${\raise0.7ex\hbox{$\pi $} \!\mathord{\left/ {\vphantom {\pi 6}}\right.\kern-0pt} \!\lower0.7ex\hbox{$6$}}$$	2.1479344	2.5899934
		$${\raise0.7ex\hbox{$\pi $} \!\mathord{\left/ {\vphantom {\pi 3}}\right.\kern-0pt} \!\lower0.7ex\hbox{$3$}}$$	2.2289891	2.5899950

**Table 3:** Different values of $\left[ {Re_{x} } \right]^{{ - \frac{1}{2}}} Nu$ for various parameters.

Fixed parameter	Parameter	Values	$\frac{{{\text{SWCNT}}/\text{H}_{2} \text{O}}}{{\left[ {Re_{x} } \right]^{{ - \frac{1}{2}}} Nu}}$	$\frac{{{\text{S + MWCNT}}/\text{H}_{2} \text{O}}}{{\left[ {Re_{x} } \right]^{{ - \frac{1}{2}}} Nu}}$
$$\begin{gathered} Br = 0.5,\gamma = 0.3,\delta_{m} = 0.2 \hfill \\ Pr = 6.2,Rd = 1,\Omega = \frac{\pi }{3},\alpha_{1} = 0.1. \hfill \\ \end{gathered}$$	$M$	0.5	2.9828435	2.9999756
		1.00	3.0954046	3.8018679
$$\begin{gathered} M = 0.5,\gamma = 0.3,\delta_{m} = 0.2 \hfill \\ Pr = 6.2,Rd = 1,\Omega = \frac{\pi }{3},\alpha_{1} = 0.03. \hfill \\ \end{gathered}$$	$Br$	0.1	2.0811574	4.0515923
		0.2	2.9920302	4.1799709
$$\begin{gathered} M = 0.5 = Br,\gamma = 0.3,\delta_{m} = 0.2 \hfill \\ Pr = 6.2,\Omega = \frac{\pi }{3},\alpha_{1} = 0.1. \hfill \\ \end{gathered}$$	$Rd$	0.3	2.9828455	3.6677423
		0.5	3.0954053	3.7829714

### Significance of velocity distribution

The variations of significant physical factors such as $M,\,\,\gamma ,\,\,\phi$ and $\Omega$ on velocity distributions $F^{\prime}\left( \xi \right)$ are portrayed in Figs. 2, 3, 4, 5. The performance of $M$ on the $F^{\prime}\left( \xi \right)$ profile for hybrid and nanocomposites (SWCNT, MWCNT) is shown in Fig. 2. It was revealed that $F^{\prime}\left( \xi \right)$ decreases as $M$ grows because it represents the ratio of electromagnetism to viscous fluid. In a magnetohydrodynamic movement the Lorentz effect is generated by the presence of $M$ which is known opposing effect. Furthermore, the movement of the nanofluid (SWCNT/H_2_O) declines quicker than the movement of hybrid nanofluid (SWCNT + MWCNT/H_2_O). The impacts of $\phi = \phi_{SWCNT} + \phi_{MWCNT}$(solid volume fraction) on $F^{\prime}\left( \xi \right)$ for hybrid and nanocomposites are highlighted in Fig. 3. The aspects of raising the volume fraction reduces the fluid $F^{\prime}\left( \xi \right)$ profile can be seen in the figure. This fact is because expanding the quantities of the volume fractional factor might produce an opposition force in the fluid motion that opposing force diminishes the fluid movement and hence the velocity of the both hybrid and nanocomposites decline. In addition, the mobility of the nanofluid (SWCNT/H_2_O) slows down faster than that of the hybrid nanofluid (SWCNT + MWCNT/H_2_O). Figure 4 illustrates the impact of the $\gamma$(slip parameter) on $F^{\prime}\left( \xi \right)$ for SWCNT + MWCNT/H_2_O and SWCNT/H_2_O nanocomposites. Figure 4 demonstrates that in both cases of hybrid nanofluids and mono fluids the fluid speed gradually decline as the magnitude of $\gamma$ parameter is elevated. This indicated that when the slip situation arise the speed of the extended surface is not the same as the velocity of the flow near the sheet. Furthermore, the nanofluid's movement (SWCNT/H_2_O) falls down more quickly than the hybrid nanofluid's (SWCNT + MWCNT/H_2_O. Figure 5 depicts the behavior of the $\Omega$(angle of inclination) on the $F^{\prime}\left( \xi \right)$ field for both cases of hybrid nanofluids and nanofluids. For increasing values of $\Omega$ it can be observed that fluid motion gradually recedes. Physically, the justification for this is that when the $\Omega$(angle of inclination) increases, the magnetic field's influence on liquid motion intensifies resulting in a drop in fluid velocity for both type of nanofluids.

### Significance of temperature distribution

In this portion the variations of important model factors such as $M,\,\,\phi ,\,\,Rd$ and $Br$ on thermal distributions $\theta \left( \xi \right)$ are depicted in Figs. 6, 7, 8, 9. Figure 6 demonstrates the influence of $M$ on $\theta \left( \xi \right)$ with SWCNT + MWCNT/H_2_O and SWCNT/H_2_O. In both cases hybrid nanoliquid and nanoliquid, the behavior of $\theta \left( \xi \right)$ profile ultimately increases for successive magnitude of $M$ as exposed in Fig. 6. Generally, when the perpendicular force on nanoparticles increases as the strength of $M$ escalate. In this situation the nanoparticles then clash with each other. The heat is produced by these collisions creates an increase in the hybrid and nanofluid temperature. Additionally, the nanofluid's temperature of hybrid nanofluid's (SWCNT + MWCNT/H_2_O) enhancing more quickly than the nanofluid's (SWCNT /H_2_O). Figure 7 exposes the influence of solid volume fraction $\phi$ on thermal $\theta \left( \xi \right)$ distribution for both cases of hybrid nanofluids and nanofluids. The $\theta \left( \xi \right)$ field of both types of fluids (hybrid and nanofluid) increases, as shown in Fig. 7. This improvement is more in SWCNT + MWCNT/H_2_O than SWCNT/H_2_O nanofluid as the quantity of $\phi$ improve. Such escalation is greater in muti wall nanocomposite than in single wall nanocomposite. Figure 8 illustrates the effect of $Rd$ on $\theta \left( \xi \right)$ profile for both cases of nano and hybrid nanofluids. For hybrid and mano nanocomposite the nature of $\theta \left( \xi \right)$ enhances with an increment in the value of $Rd$. It is observed from the sketch that hybrid nanoliquid has a sluggish heating dispersion pattern than nanoliquid. From a physical perspective when the value of $Rd$ grows progressively, the mean absorption coefficient drops and diffusing flux happen as a consequence of the temperature profile that enhances the heat range. Figure 9 explores the diversity of $\theta \left( \xi \right)$ profile versus the enhanced quantity of $Br$ using both types of fluids. This chart illustrates that when the values of $Br$ goes up then the behavior of $\theta \left( \xi \right)$ increases significantly. This show that flow pattern of SWCNT/H_2_O liquid has a faster $\theta \left( \xi \right)$ distribution than SWCNT + MWCNT/H_2_O. Physically, by reducing the heat conduction because of the dissipation is associated with higher values of $Br$. As a result, the internal energy of fluids rises.

### Significance of entropy optimization and Bejan number

In this section the variations of some substantial physical variables like $M,\,\,\alpha_{1} ,\,\,Br$ and $Rd$ on entropy generation profile $NG$ are portrayed in Figs. 10, 11. Figure 10 demonstrates the aspects hybrid and nanofluid of $\alpha_{1}$(temperature differential parameter) on $NG$. This diagram shows a declining impact with hybrid and mano nanocomposite. Also, the $NG$ profile of hybrid nanofluids (SWCNT + MWCNT/H_2_O) raises more quickly as compared to the nanofluids (SWCNT/H_2_O). but, in the surrounding liquid its show negligible effect. The effect of the $Br$ on the $NG$ is visualized in Fig. 11 in presence of SWCNT + MWCNT/H_2_O and SWCNT/H_2_O nanofluids. In this case, the $NG$ increases with respect to the greater $Br$ estimations. Physically, $Br$ is a source of heat production in the liquid circulating domain. The $NG$ is improved by incorporating energy created with energy transformation from the wall of surface. As the $Br$ rises the heat conductivity of fluid declines leading to an improvement in $NG$. Figure 12 displays the effect of $Rd$ on $NG$ for SWCNT + MWCNT/H_2_O and SWCNT/H_2_O nanoliquid. Through thermal radiation, entropy degrades. The graph illustrates that for both types of nanofluid $NG$ expands rapidly as a result to rising improving the values of $Rd$. Also, we note that SWCNT + MWCNT/H_2_O has a speedier nature of $NG$ than SWCNT/H_2_O.

In this portion the variations of important model factors such as $M,\,\,\alpha_{1} ,\,\,Br$ and $Rd$ on $Be$ are depicted in Figs. 13, 14. Figure 11 reveals the impact of $M$ on $Be$ profile for hybrid and mano nanocomposite. In both hybrid and nanoliquid scenarios as perceived in Fig. 13, the $Be$ profile behavior eventually increases for successive values of $M$. Figure 14 highlighted the characteristics of hybrid and nanofluid of $Rd$ on $Be$. This diagram shows an improving pattern for both SWCNT + MWCNT/H_2_O and SWCNT/H_2_O. Also, we noted that the $Be$ profile of hybrid nanofluids (SWCNT + MWCNT/H_2_O) raises more quickly as compared to the nanofluids (SWCNT/H_2_O).

### Significance of drag force and heat transfer rate

**Figure 15:**
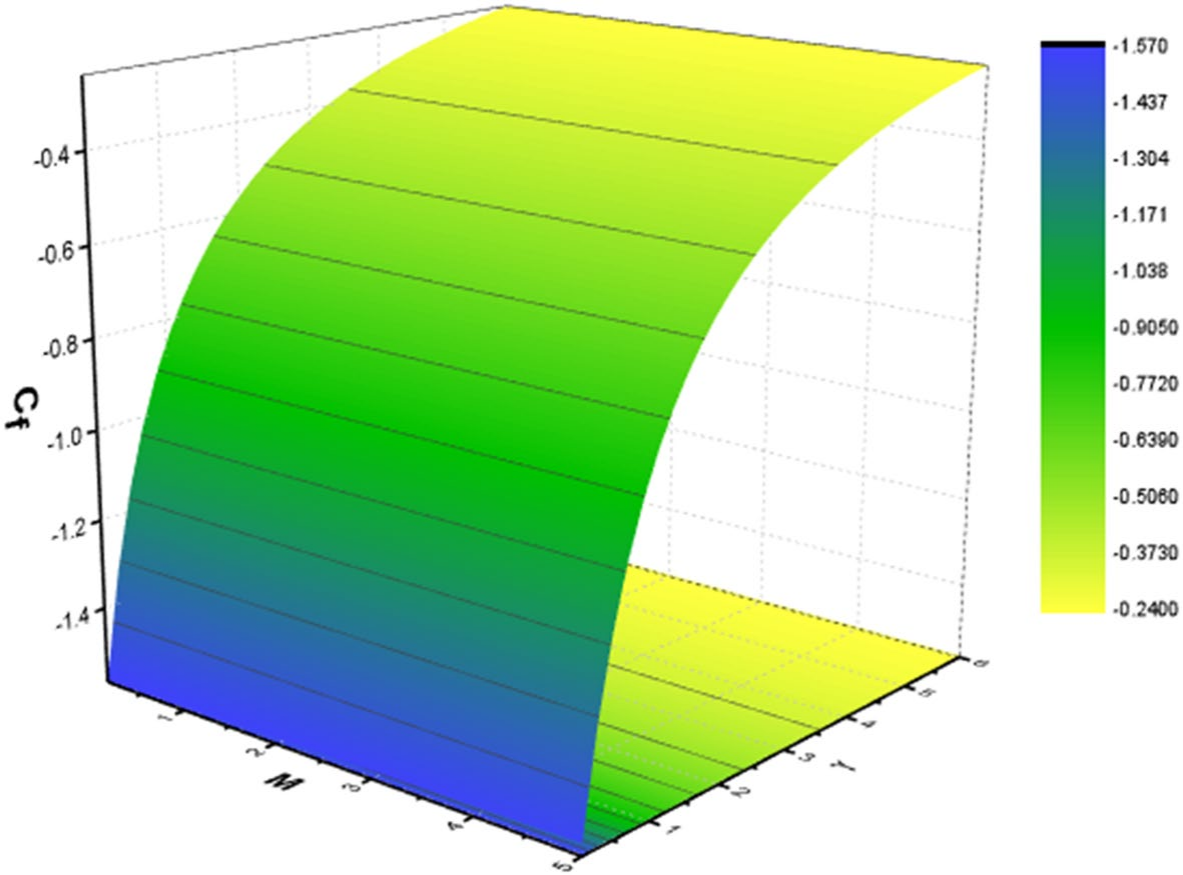
Behavior of $\left[ {Re_{x} } \right]^{0.5} C_{f}$ for *M* and $\gamma$.

**Figure 16:**
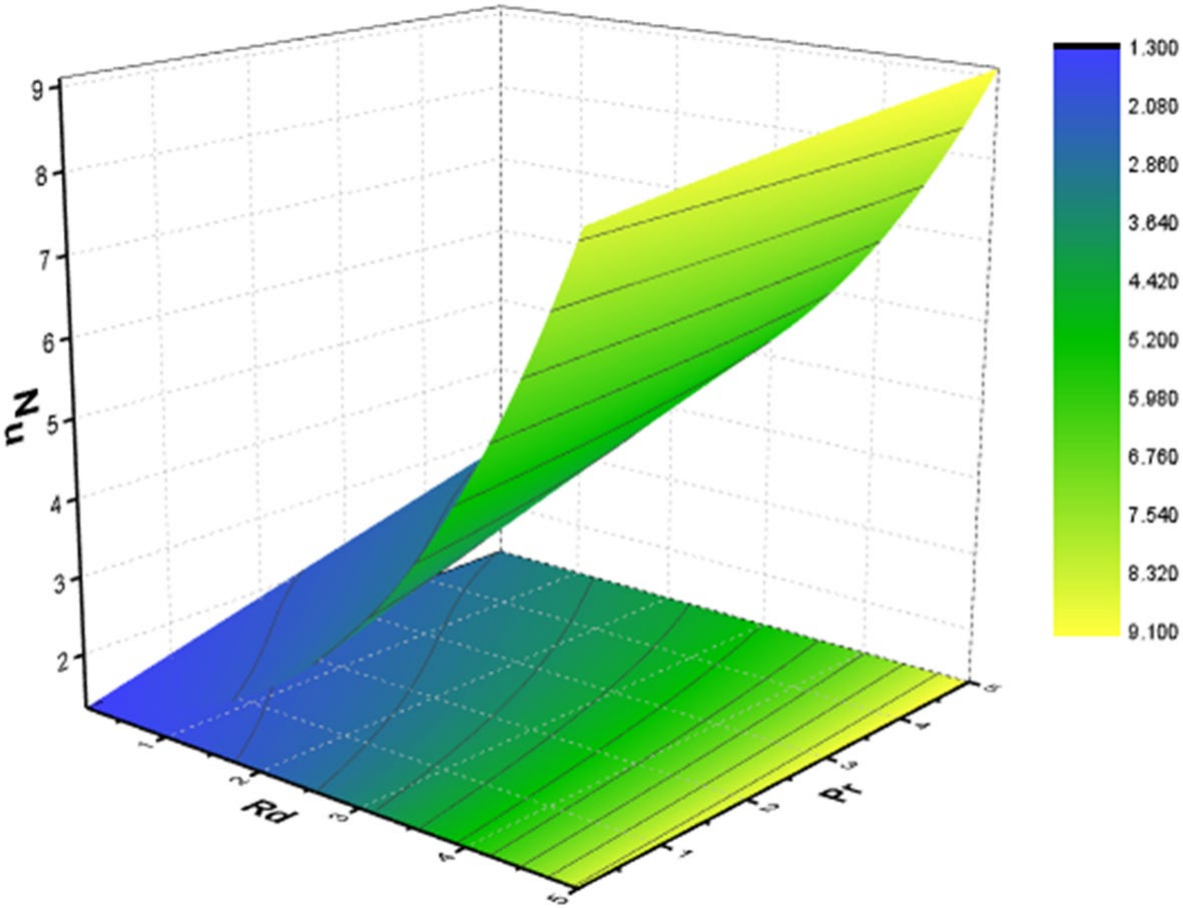
Behavior of $\left[ {Re_{x} } \right]^{ - 0.5} Nu$ for *Rd* and $Pr$.

**Figure 17:**
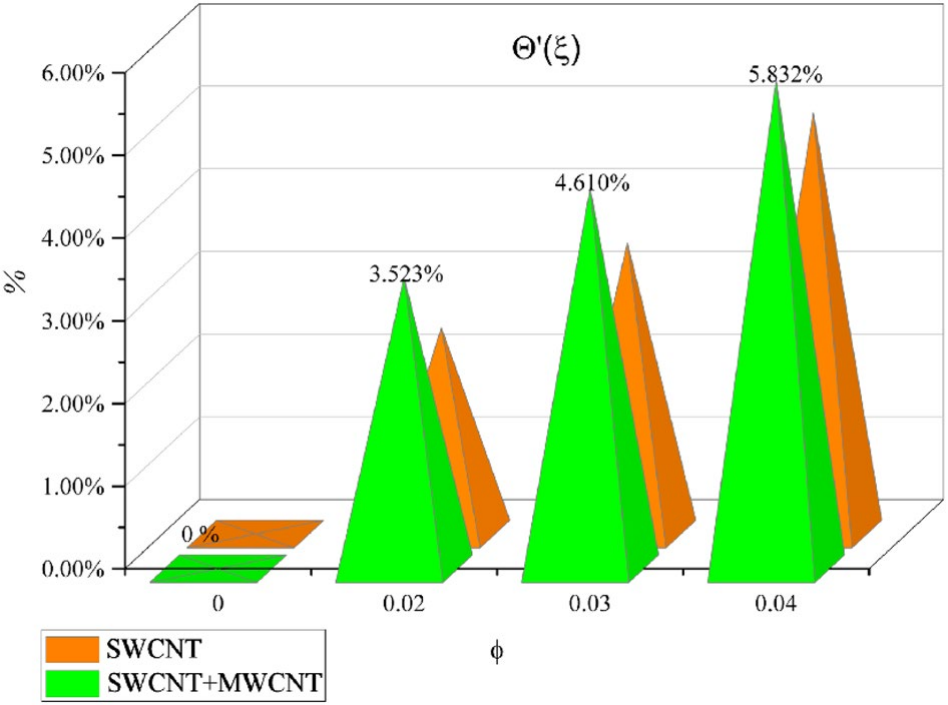
Percentage enhancement in $\left[ {Re_{x} } \right]^{ - 0.5} Nu$ for $\phi$.

**Figure 18:**
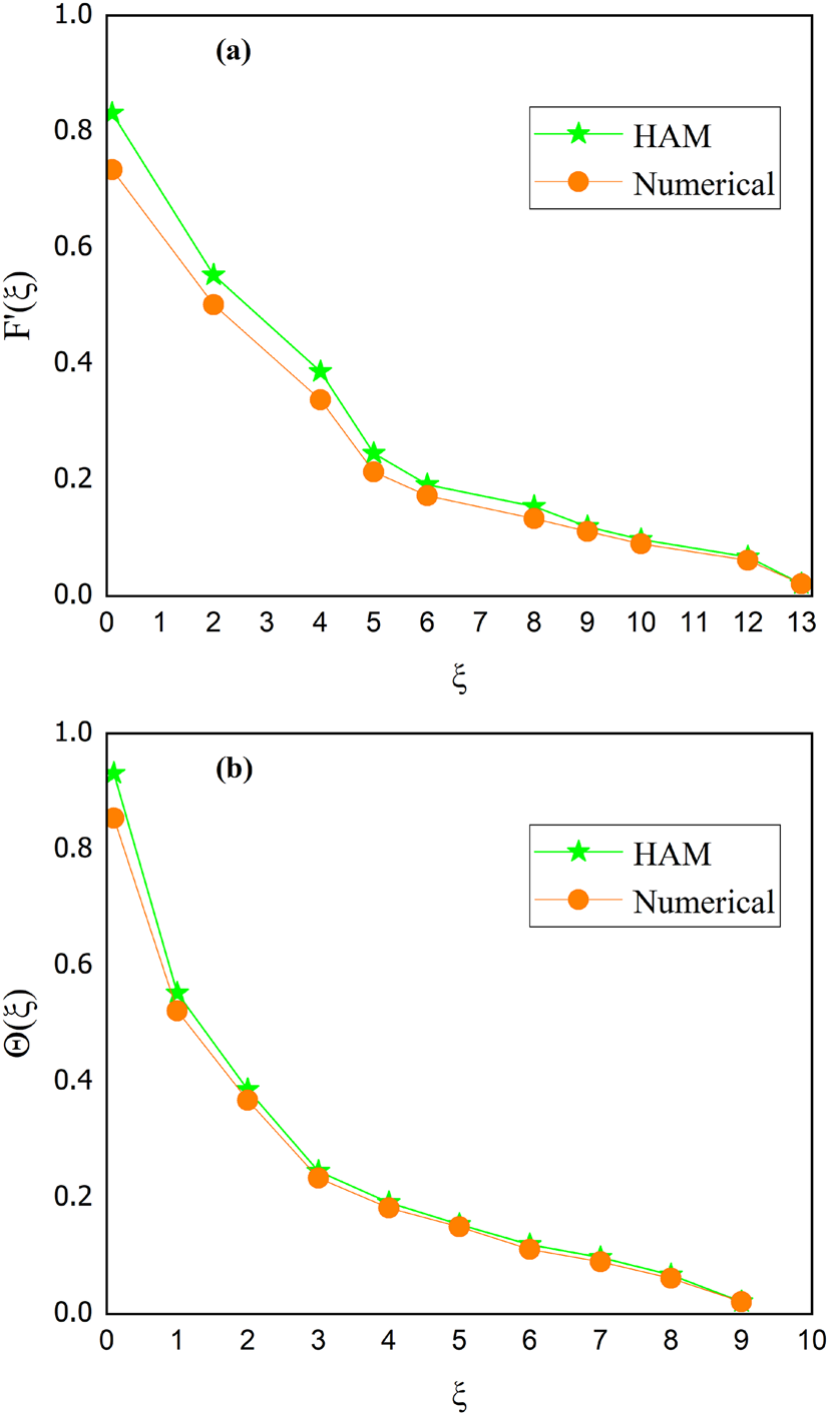
Validation of HAM and numerical technique (shooting) for (**a**) velocity profile (**b**) temperature profile.

Tables 2 and 3 are mathematically arranged to examine the variability of features ($C_{f}$ and $Nu$) for SWCNT/H_2_O and SWCNT + MWCNT/H_2_O respectively. According to Table 2, skin friction is ramped up by the $M,\,\,\gamma$ but decline for high values of $\Omega$. The $Nu$ jumps with higher values of $M,\,\,Br$ and $Rd$ as seen in Table 3. The current outcomes are verified using published data using a restricting strategy see Table 4 to verify the accuracy of mathematical calculation and are determined to be acceptable. Figure 15 show the 3D charts of $C_{f}$. It has been found that when the values of $\delta_{m} ,\,\,\gamma$ grow the $C_{f}$ decreases. The 3D results of $Nu$ for parameters $Rd$ and $Pr$ are shown in Fig. 16. Figure 17 displays the percent improvement in the heat flux for mano and hybrid nanofluid. This graph demonstrates that when hybrid nanocomposite is used instead of simple nanofluids, the rate of heat transmission is usually enhanced. Figure 18a,b show the comparison between the HAM and the numerical technique (shooting method) for the fluid velocity and thermal profiles. After performing some iterations, a closed agreement has been obtained from these two procedures.

**Table 4:** Numerical outcomes compared with^34,35^ of $\left[ {Re_{x} } \right]^{0.5} C_{f}$ for various parameters.

$M$	$\delta_{m}$	$\gamma$	^34^	^35^	Current work
1.0	0.5	0.5	1.223676	1.2236758	1.2236756
1.2	0.5	0.5	1.412204	1.4122036	1.4122038
1.5	0.5	0.5	1.64600	1.6464991	1.6464995
2.0	0.5	0.5	1.765886	1.7658849	1.7658850
1.5	0.0	0.5	1.395644	1.3956438	1.3956441
1.5	0.2	0.5	1.494254	1.4942535	1.4942539
1.5	0.5	0.5	1.646600	1.6465991	1.6464995
1.5	0.5	0.0	0.967329	0.9673285	0.9673283
1.5	0.5	0.2	1.278297	1.2781968	1.2781970
1.5	0.5	0.5	1.646600	1.6465991	1.6464995
1.5	0.5	0.7	2.186934	2.1869332	2.1869337

## Conclusions

Using an exponentially stretching surface, we want to investigate the effect of slanted MHD on radiative fluxes of Maxwell nanofluid embedded with CNT nanomaterials. Entropy production is calculated using the second rule of thermodynamics. Moreover, Ohmic heat, dissipations, thermal radiation and velocity slip constraints are described in the governing formulations. Analytically, the reduced mathematical formulation is estimated. Here a brief overview of our main insights:

- For both types of fluids (SWCNT + MWCNT/H_2_O and SWCNT/H_2_O), the liquid velocity falls as the $M,\,\,\gamma ,\,\,\phi$ and $\Omega$ values increase.
- By incorporating nanocomposites into the base fluid, the thermal characteristics are modified, and it also assists in regulating the mobility of the nanofluids.
- The raising of $M,\,\,Rd$ and $Br$ values is boosted the thermal fields in both cases (hybrid and simple nanofluid).
- Although slipperiness slows the liquid transmission and lets to reduction in velocity and temperature fields, it is noteworthy to note that enhancing the slip factor minimizes overall entropy.
- For increasing values of $M,\,\,Rd$ and $Br$ the rate of heat transmission accelerates.
- The drag force maximizes as the value of $M,\,\,\gamma$ grows while it deteriorates as the value of $\Omega$ reduces.
- The higher measurement of $Br$ and $Rd$ reflect to rise the entropy production rate.
- Bejan number shows the enhancing behavior for high $Rd$ and $M$.
- We obtained a significant agreement when we compared our results to existing work.
- The research described in this publication can be expanded to include the examination of more non-Newtonian nanofluids in several situations under varied conditions.

## Data Availability

The data that support the findings of this study are available from the corresponding author upon reasonable request.
